# The Respiratory Rate-Oxygenation Index predicts failure of post-extubation high-flow nasal cannula therapy in intensive care unit patients: a retrospective cohort study

**DOI:** 10.5935/0103-507X.20220477-en

**Published:** 2022

**Authors:** Yuli V. Fuentes, Katherine Carvajal, Santiago Cardona, Gina Sofia Montaño, Elsa D. Ibáñez-Prada, Alirio Bastidas, Eder Caceres, Ricardo Buitrago, Marcela Poveda, Luis Felipe Reyes

**Affiliations:** 1 Universidad de La Sabana - Bogotá, Colombia.; 2 Clínica Universidad de La Sabana - Chía, Colombia.; 3 Hospital Pablo Tobón Uribe y Clínica Sagrado Corazón - Medellin, Colombia.; 4 Fundación Clínica Shaio - Bogota, Colombia.

**Keywords:** Cannula, Oxygenation, Respiratory rate, Airway extubation, Pneumonia, Critical care, Intensive care units

## Abstract

**Objective:**

To investigate the applicability of the Respiratory Rate-Oxygenation Index to identify the risk of high-flow nasal cannula failure in post-extubation pneumonia patients.

**Methods:**

This was a 2-year retrospective observational study conducted in a reference hospital in Bogotá, Colombia. All patients in whom post-extubation high-flow nasal cannula therapy was used as a bridge to extubation were included in the study. The Respiratory Rate-Oxygenation Index was calculated to assess the risk of post-extubation high-flow nasal cannula failure.

**Results:**

A total of 162 patients were included in the study. Of these, 23.5% developed high-flow nasal cannula failure. The Respiratory Rate-Oxygenation Index was significantly lower in patients who had high-flow nasal cannula failure [median (IQR): 10.0 (7.7 - 14.4) versus 12.6 (10.1 - 15.6); p = 0.006]. Respiratory Rate-Oxygenation Index > 4.88 showed a crude OR of 0.23 (95%CI 0.17 - 0.30) and an adjusted OR of 0.89 (95%CI 0.81 - 0.98) stratified by severity and comorbidity. After logistic regression analysis, the Respiratory Rate-Oxygenation Index had an adjusted OR of 0.90 (95%CI 0.82 - 0.98; p = 0.026). The area under the Receiver Operating Characteristic curve for extubation failure was 0.64 (95%CI 0.53 - 0.75; p = 0.06). The Respiratory Rate-Oxygenation Index did not show differences between patients who survived and those who died during the intensive care unit stay.

**Conclusion:**

The Respiratory Rate-Oxygenation Index is an accessible tool to identify patients at risk of failing high-flow nasal cannula post-extubation treatment. Prospective studies are needed to broaden the utility in this scenario.

## INTRODUCTION

Acute hypoxic respiratory failure (AHRF) is the leading cause of admission to intensive care units (ICUs) worldwide, with an associated mortality of 52%.^(1)^ The cornerstone of AHRF treatment is mechanical ventilatory support. Invasive mechanical ventilation (IMV) is the most frequent strategy of respiratory support in patients admitted to the ICU due to AHRF. IMV is used to improve oxygen delivery and ventilation and reduce the work of breathing in multiple clinical conditions. Despite its proven utility, patients treated with IMV may develop several complications, including barotrauma, hospital-acquired infections, sedation-related adverse effects, difficult weaning, deconditioning, *delirium*, and extubation failure, among others.^(1,2)^ It is known that even patients with elective extubation have a 14% failure rate within the first 48 to 72 hours, which is associated with increased mortality.^(3)^ Several scores and strategies have been used to identify patients at higher risk of reintubation due to failure, to identify patients needing closer monitoring during the extubation period, and/or requiring less invasive ventilatory support.^(4-6)^

High-flow nasal cannula (HFNC) is a form of oxygen therapy that provides high flow oxygen up to 60L/minute, conditioned to adequate temperature and humidity. It has emerged as a promising therapy to treat patients with hypoxemic respiratory failure.^(4)^ High-flow nasal cannula improves oxygenation parameters by decreasing airway dead space, reducing oxygen dilution, and providing positive air pressure.^(4-7)^ A HFNC can also offer an inspired oxygen fraction (FiO_2_) between 21% and 100%. This therapy has proven helpful for different pathologies and clinical scenarios, such as patients with AHRF, ventilatory support during bronchoscopy studies, hypoxemia due to severe heart failure,^(8)^ and bridge therapy after extubation.^(3,5)^ Regarding the use of HFNC in patients during the post-extubation period, a meta-analysis concluded that HFNC is an efficient and reliable alternative to decrease the risk of reintubation compared to conventional oxygen therapy.^(3,5)^ Despite the advantages of HFNC treatment in extubated patients with AHRF, some still require reintubation, which increases their morbidity and mortality.^(9-11)^

The Respiratory Rate-Oxygenation (ROX) Index, defined as the ratio of oxygen saturation to a fraction of inspired oxygen (SpO_2_/FIO_2_) with the respiratory rate, was validated in patients with AHRF due to pneumonia.^(12)^ Regarding community-acquired pneumonia (CAP), the index was efficient in predicting patients with HFNC with a worse prognosis and requiring IMV as a primary ventilatory support strategy. Patients with a ROX index > 4.88 likely had better clinical outcomes with HFNC treatment. In comparison, those patients with a ROX index < 4.88 were at a higher risk of requiring IMV and developing worse clinical outcomes.^(6)^

Importantly, it is unknown whether the ROX index may predict extubation failure and clinical outcomes in patients treated with HFNC as bridge therapy (i.e., during post-extubation). Therefore, this study aimed to investigate the applicability of the ROX index in this scenario. We hypothesize that the ROX index will predict a higher risk of reintubation in ICU patients diagnosed with AHRF who were treated with HFNC after extubation. To test this hypothesis, we assessed the ROX index in patients with a high risk for extubation failure who received HFNC treatment after the IMV was withdrawn.

## METHODS

### Study design

This observational retrospective study was carried out in a tertiary hospital in Bogotá, Colombia. This study included patients hospitalized in the ICU who required IMV support and were treated with HFNC in the post-extubation period between 2016 and 2018. During this period, demographic, laboratory, predictive, and severity scores (e.g., Tobin Index, Acute Physiology and Chronic Health Evaluation - APACHE and Sequential Organ Failure Assessment - SOFA) and hemodynamic data were documented from admission to ICU discharge or until death (Tables 1 and 2). The ethics committee of the institution approved the study. Due to the nature of the study, informed consent was not required.

**Table 1:** Baseline patients' characteristics

Characteristic	Optiflow fail n = 38	Optiflow no fail n = 124	p value
Demographic			
Age (years)	67.6 ± 18.0	65.2 ± 16.6	0.73
Weight (kg)	68.8 ± 13.4	68.0 ± 12.6	0.34
Height (m)	1.6 ± 0.1	1.6 ± 0.1	< 0.1
BMI (kg/m^2^)	25.9 ± 4.8	25.2 ± 4.5	0.84
Comorbid conditions			
Tobacco use	10 (26.3)	36 (29)	0.74
COPD	10 (26.3)	49 (39.5)	0.13
Pulmonary hypertension	7 (18.4)	43 (34.7)	0.05
Diabetes mellitus	14 (36.8)	38 (30.6)	0.47
Arterial hypertension	26 (68.4)	84 (67.7)	0.93
Heart failure	20 (52.6)	68 (54.8)	0.81
Chronic kidney disease	9 (23.7)	29 (23.4)	0.97
Hepatic disease	1 (2.6)	1 (0.8)	0.37
Obesity	5 (13.2)	19 (15.3)	0.74
HIV	0 (0)	1 (0.8)	0.57
Other immunosuppression	1 (2.6)	17 (13.7)	0.05
Ischemic heart disease	10 (26.3)	39 (31.5)	0.54
Pulmonary interstitial disease	0 (0)	8 (6.5)	0.10
OSA	5 (13.2)	7 (5.6)	0.12
Medical treatment before admission			
Statin	12 (31.6)	56 (45.2)	0.13
ACEi	17 (44.7)	52 (41.9)	0.76
Beta-blockers	18 (47.4)	48 (38.7)	0.34
Corticoid	11 (28.9)	46 (37.1)	0.35
Ipratropium bromide	7 (18.4)	24 (19.4)	0.89
Salbutamol	2 (5.3)	11 (8.9)	0.47
Clinical diagnosis at admission			
ARDS	1 (2.6)	2 (1.6)	0.68
Acute pulmonary thromboembolism	0 (0)	15 (12.1)	0.02
Post cardiac arrest syndrome	1 (2.6)	7 (5.6)	0.45
Acute coronary syndrome	4 (10.5)	14 (11.3)	0.89
Acute heart failure	10 (26.3)	23 (18.5)	0.29
VAP	3 (7.9)	9 (7.3)	0.89
CAP	15 (39.5)	26 (21)	0.02
HAP	2 (5.3)	2 (1.6)	0.20

BMI - body mass index; COPD - chronic obstructive pulmonary disease; HIV - human immunodeficiency virus; OSA - obstructive sleep apnea; ACEi - angiotensin-converting enzyme inhibitors; ARDS - acute respiratory distress syndrome; BUN - blood urea nitrogen; VAP - ventilator acquired pneumonia; CAP - community-acquired pneumonia; HAP - hospital acquired pneumonia. The results are expressed as the mean ± standard deviation or n (%).

**Table 2:** Inpatient admission characteristics

Characteristic	Optiflow fail n = 38	Optiflow no fail n = 124	p value
Severity score			
APACHE score	11.8 ± 4.7	9.5 ± 3.8	3.02
SOFA score	6.9 ±3.3	6.0 ± 2.5	1.88
Pre-extubation score			
Tobin score	37.4 ± 14.4	40.6 ± 15.8	< 0.9
Physiologic measures at admission			
Heart rate (bpm)	85.0 ± 14.6	81.0 ± 15.1	1.42
Respiratory rate (bpm)	22.6 ± 6.5	19.2 ± 4.6	3.64
Systolic pressure (mmHg)	124.1 ± 21.4	123.2 ± 18.5	0.26
Mean arterial pressure (mmHg)	86.8 ± 15.1	86.2 ± 13.7	0.22
Oxygen saturation (%)	90.2 ± 7.2	91.3 ± 5.0	< 0.9
Glasgow score	14.4 ± 0.8	14.5 ± 1.0	< 0.1
Creatinine (mg/dL)	1.6 ± 1.2	1.4 ± 1.5	0.62
BUN (mg/dL)	42.7 ± 17.7	33.5 ± 15.9	3.03
Hemoglobin (g/dL)	11.2 ± 2.4	11.1 ± 2.2	0.20
Platelets/mm^3^	204684 ± 93012	204774 ± 109258	< 0.1
Procalcitonin (ng/mL)	7.5 ± 10.8	26.0 ± 91.3	< 0.1
Troponin (ng/mL)	3400.1 ± 5851.7	12277.38 ± 20252.3	< 0.1
pH	7.4 ± 0.1	7.4 ± 0.1	< 0.1
PaCO_2_ (mmHg)	39.8 ± 12.4	37.4 ± 9.3	1.21
PaO_2_ (mmHg)	74.5 ± 16.6	74.4 ± 20.7	0.03
HCO_3_- (mmol/L)	24.7 ± 5.2	24.9 ± 4.6	< 0.1
Lactate (mmol/L)	1.9 ± 1.6	1.6 ± 0.6	1.82
PaO_2_/FiO_2_	193.6 ± 68.0	192.6 ± 56.2	0.09

APACHE - Acute Physiology and Chronic Health Evaluation; SOFA - Sequential Organ Failure Assessment; BUN - blood urea nitrogen; PaCO_2_ - partial pressure of carbon dioxide; PaO_2_ - partial pressure of oxygen; HCO_3_ - bicarbonate; PaO_2_/FiO_2_ - ratio of partial pressure arterial oxygen and fraction of inspired oxygen. The results are expressed as the mean ± standard deviation.

### Participants

The inclusion criteria were patients admitted to the postoperative and non-postoperative ICUs treated with invasive ventilatory support for at least 24 hours due to AHRF and receiving HFNC therapy immediately after extubation. All pathologies associated with the requirement for IMV were included (acute respiratory distress syndrome, acute pulmonary thromboembolism, post-cardiac arrest syndrome, acute coronary syndrome, acute heart failure, ventilator-acquired pneumonia, CAP, and hospital-acquired pneumonia). No limit for IMV days was applied. The ICU team decided to extubate according to clinical criteria and the international weaning protocol guidelines. All patients were classified as high risk for reintubation, defined as those older than 65 years, smokers, with the presence of chronic obstructive pulmonary disease or another comorbid condition. Additionally, a previous failure in an extubation attempt or history of a negative weaning test (spontaneous breath test through pressure support ventilation mode, leak test, or airway score) were criteria for high-risk definition. Patients who fulfilled the inclusion criteria were included in the analysis. Patients under 18 years of age and those who required intubation for diagnostic or therapeutic procedures were excluded.

### Weaning protocol

As soon as the medical reason for mechanical ventilation was resolved and patients were hemodynamically and neurologically stable, they became eligible for a spontaneous breath test (SBT). Mechanical ventilator parameters were adjusted for selected patients (pressure support - PS = 0cmH_2_O, positive end-expiratory pressure - PEEP = 0cmH_2_O, and FIO_2_ < 50%). If they did not develop clinical signs of respiratory distress and did not vary > 50% in ventilatory values (tidal volume) or vital signs, they were considered to pass the SBT (positive test) and were fit for extubation. When changes were assessed, they were not considered for extubation, were returned to previous mechanical ventilation parameters, and were submitted to a new assessment after 24 hours. Additionally, the cuff-leak test was used as a simple method to predict the occurrence of post-extubation stridor. This test was performed by cuff deflation and measuring the expired tidal volume a few breaths later. A negative test was considered in patients whose leakage was small and had laryngeal stridor; therefore, they were not extubated. Additionally, the Coplin test^(13)^ was used to assess airway protection by evaluating pharyngeal reflex, cough quality, and sputum characteristics. A positive test was defined as a score < 7, which predicted extubation success. Due to the heterogeneity and variability in predictive values of each weaning test, all tests were simultaneously carried out to decide the best candidate for extubation.

Immediately after extubation, patients received a bridge strategy using HFNC (i.e., Optiflow, Fisher & Paykel). The FiO_2_ was titrated according to Bogotá altitude to obtain an oxygen saturation higher than 92%. The flow was adjusted according to patient tolerance. It was considered that the maximum tolerated flow was obtained in the first 10 minutes of treatment. No other methods of intermittent ventilatory support were used with HFNC therapy (e.g., noninvasive ventilation - NIV). ROX index validated by Roca et al.^(6)^ was calculated 4 - 6 hours after establishing HFNC support. Extubation failure was defined as the inability to tolerate removal of IMV and the need for reintubation within 72 hours after extubation because of hypoxemia (PaO_2_ < 60mmHg), non-permissible hypercapnia (PaCO_2_ > 60mmHg with pH < 7.2), or labored breathing.

### Clinical outcomes

The primary outcome of this study was to determine if the ROX index can identify the risk of extubation failure in patients treated with HFNC as bridge therapy. We determined whether the ROX index could predict ICU mortality as a secondary outcome.

### Statistical analysis

A retrospective collection of data was made, and those records with missing data greater than 20% were excluded. Qualitative variables are summarized as frequencies and percentages. For numerical variables, if their distribution was normal, the mean and standard deviation (SD) were used. In cases of nonnormal distribution, median and interquartile ranges (IQR) were calculated and reported. We used Fisher’s exact test to compare categorical variables and the nonparametric test (Mann-Whitney U Test) to evaluate continuous variables. The ROX index was categorized at a threshold of 4.88 to calculate the odds ratio (OR) of extubation failure. Logistic regression analysis was performed using age, sex, respiratory disease comorbidity, Glasgow score, and pH values as independent variables. Receiver operating characteristic (ROC) curves were calculated using the ROX index and extubation failure (outcome). A statistical significance of 0.05 and confidence intervals of 95% (95%CI) was chosen. All statistical analyses were performed using IBM Statistical Package for the Social Sciences (SPSS), version 27.0. Armonk, NY: IBM Corp.

## RESULTS

A total of 162 patients treated with HFNC after extubation were included in the study. A total of 23.5% (38/162) of the cohort had extubation failure despite the use of HFNC, and 76.5% (124/162) responded adequately to bridge therapy. Both patients who failed and those who did not fail showed similar characteristics as the mean (SD) age [67.6 (18.0) *versus* 65.2 (16.6); p = 0.73] and body mass index (BMI) [25.9 (4.8) *versus* 25.2 (4.5); p = 0.84]. Overall mortality during the ICU stay was 17.3% (28/162) (Table 1).

There were no significant differences regarding comorbidity conditions. Immunosuppression [2.6% (1/38) *versus* 13.7% (17/124); p = 0.05] and pulmonary hypertension [18.4% (7/38) *versus* 34.7% (43/124); p = 0.05] were the characteristics that showed the most distant proportions between the groups. Additionally, there were no significant differences in medical treatment before admission between patients who had or did not have HFNC failure. Community-acquired pneumonia was the most frequent diagnosis at admission in patients who had extubation failure on HFNC compared with patients who did not fail [39.5% (15/38) *versus* 21% (26/124); p = 0.02].

Severity scores at admission measured before extubation were similar in both groups, with a mean (SD) APACHE score of 11.8 (4.7) *versus* 9.5 (3.8); p = 3.02 and SOFA score of 6.9 (3.3) *versus* 6.0 (2.5); p = 1.88. Additionally, the Tobin score did not show significant differences [37.4 (14.4) *versus* 40.6 (15.8); p < 0.9]. The only physiologic parameter that differed between the groups was arterial oxygen pressure (PaO_2_), with a higher mean (SD) in patients who had HFNC failure than in those who did not [74.5mmHg (16.6) *versus* 74.4mmHg (20.7); p = 0.03]. The complete physiological admission parameters are described in table 2.

**Figure 1:**
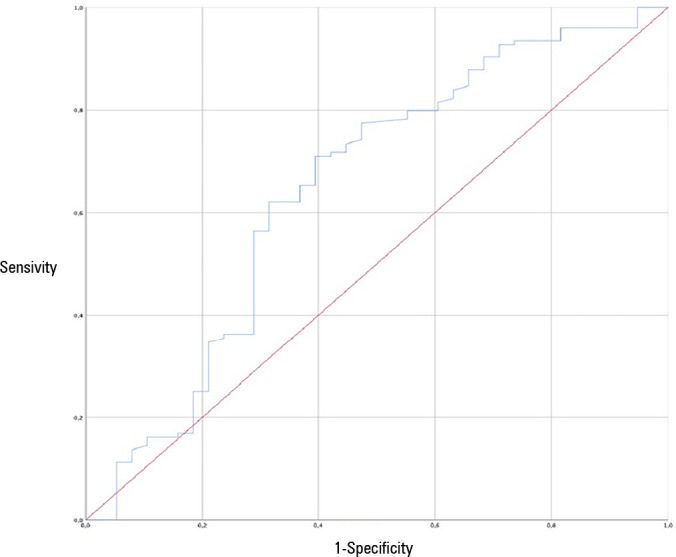
Receiver operating characteristic curve of the Respiratory Rate-Oxygenation Index predictive value.

The ROX index was statistically lower in patients who had HFNC failure than in those who tolerated bridge therapy [median (IQR): 10.0 (7.7 - 14.4) *versus* 12.6 (10.1 - 15.6), p = 0.006] (Table 3). The ROX index was categorized by a cutoff point of 4.88. The crude risk of extubation failure showed an OR of 0.23 (95%CI: 0.17 - 0.30) and was stratified by severity and comorbidity. The adjusted OR of reintubation for a ROX index > 4.88 was 0.89 (95%CI 0.81 - 0.98). After logistic regression analysis for HFNC therapy failure, the ROX index had an adjusted OR of 0.90 (95%CI: 0.82 - 0.98, p = 0.026). In terms of the predictive capacity of the ROX index for extubation failure, the area under the ROC curve was 0.64 (95%CI: 0.53 - 0.75, p = 0.06) (Figure 1). Finally, the median (IQR) ROX index did not show significant differences between patients who survived and those who died during the ICU stay [11.6 (7.8 - 16.5) *versus* 12.4 (10.0 - 15.2); p = 0.3] (Table 3).

**Table 3:** ROX score in patients who had high-flow nasal cannula failure and died during intensive care unit stay

Outcomes	ROX score	p value
Optiflow fail	10 (7.7 - 14.4)	0.006
Optiflow no fail	12.6 (10.1 - 15.6)	
Survival	11.6 (7.8 - 16.5)	0.30
Mortality	12.4 (10.0 - 15.2)	

Results expressed as median (interquartile range).

## DISCUSSION

This study found that approximately a quarter of patients admitted to the ICU, receiving HFNC as bridge therapy posterior to IMV requirement, developed extubation failure. Notably, the ROX index was a valuable tool to determine HFNC failure in extubated patients. Then, patients with a low ROX index had a higher adjusted risk of reintubation. Finally, this score did not identify patients at higher risk of dying in the ICU.

Among ventilatory supports available for CAP patients, HFNC is an alternative widely described.^(8,14,15)^ Additionally, in patients who receive invasive mechanical ventilation, oxygen therapy with HFNC allows an optimal transition in the post-extubation period, especially for those at high risk of extubation failure. Despite this, some patients still experience reintubation.^(5,16,17)^ Xu et al. developed a systematic review and meta-analysis from randomized controlled trials (RCTs) of patients who received HFNC after extubation. From eight RCTs, they estimated an overall extubation failure rate of 12.9% (108/839) within 72 hours after HFNC use.^(18)^ Another RCT from Thille et al. described a reintubation rate of 18.2% (55/302) on Day 7.^(19)^ In the last year, Kansal et al. reported in a multicenter observational study that 16.8% (41/244) of their extubated patients had HFNC failure ≤ 7 days.^(20)^ However, detailing the baseline characteristics of the above patients, our patients had higher SOFA scores and more compromised ventilatory laboratories, which indicates that our cohort included patients with greater severity, which could be why our rate of HFNC failure was higher. These previous results support our findings and highlight the health burden of these complications and the importance of finding an early predictor of HFNC treatment failure in extubated patients.

The ROX index is a representative tool used in patients with HFNC to assess the work of breathing. Community-acquired pneumonia patients with a ROX index greater than or equal to 4.88 after 2, 6, and 12 hours of HFNC therapy were less likely to develop respiratory failure.^(6,12)^ Hill et al. proposed new scenarios for applying this strategy, including for patients with a high risk of extubation failure.^(21)^ Goh et al. developed a prospective cohort study of 46 extubated patients and found that the ROX index was lower in those who failed HFNC. Additionally, they described an adjusted HR of 0.17 (0.03 - 0.83) for a cutoff point > 7.0 at 24 hours in the Cox regression analysis. However, this study had a very small sample size; therefore, the confidence intervals were wide, and no differences were found, with a cutoff point of 4.88.^(22)^ Additionally, in a retrospective study, Lee et al. included 276 extubated patients and evidence of an unadjusted HR of 0.37 (0.16 - 0.81) at 12 hours with a ROX index cutoff point > 10.4 for the risk of reintubation. Additionally, they described an AUC of 0.72 (0.66 - 0.78) for predicting the success of HFNC.^(23)^ Literature on this particular scenario is scarce, and our results agreed with the utility of the ROX index in the post-extubation period, favoring a narrower cutoff point of 4.88, unlike other authors who were laxer with their indices.

Reintubation is a common complication in ICU ventilated patients, and its impact on morbidity and mortality is high. The mortality rate associated with extubation failure is between 30 and 40%,^(24)^ and consequently, patients who fail HFNC after invasive mechanical ventilation removal present significantly higher mortality events than patients who tolerate HFNC treatment.^(23,25)^ To improve survival, a predictor is necessary that allows early decisions and does not delay reintubation in patients who fail HFNC.^(22,26)^ The ROX index has also been studied for mortality prediction in patients with AHRF at the emergency department or the intermediary care unit. In these patients, a high ROX index (7.0 or higher) was a protective factor for mortality in the ICU and at 28 or 30 days.^(27-29)^ However, there is no evidence that relates the ROX index in extubated patients who receive HFNC as a bridge therapy and mortality rates. Our study did not find significant differences between the ROX index in patients who died during the ICU stay and those who survived. It is necessary to develop more studies to determine if the ROX index predicts ICU mortality in these patients.

Our study has some limitations and strengths that are important to acknowledge. This study was from a retrospective cohort carried out in a single center and had a small sample size, limiting the generalizability of the results and statistical power. However, our cohort had a balanced sample that included medical and surgical patients with similar laboratory and clinical parameters, such as comorbidities, arterial gases, and severity, among others. Second, we acknowledge the risk of information bias due to our retrospective design based on medical records. Nevertheless, different strategies were used to prevent bias during the methodology and statistical analysis, such as double validation conducted by other investigators and logistic regression analysis that was adjusted to control confounding variables and reduce other risks of bias. Third, some unmeasured variables were not included in the analysis, such as individual parameters of HFNC therapy (flow, FiO_2_, and temperature), associated adverse events, time of invasive ventilatory support, and ROX score calculated at different time points, which limits the scope of this study. Despite this, our study answers the questions posed initially. It provides novel information not currently available in the literature, developing new hypotheses to be answered in future studies and making it valuable.

## CONCLUSION

Extubation failure is a frequent complication of intensive care unit patients. A Respiratory Rate-Oxygenation Index < 4.88 is an easy-to-use score that could identify patients at higher risk of high-flow nasal cannula failure during post-extubation treatment. Prospective studies are needed to confirm the utility of this index in intensive care unit patients treated with high-flow nasal cannula as a bridge therapy and the relationship between the Respiratory Rate-Oxygenation Index and intensive care unit mortality.
